# P-2086. Knowledge of Antimicrobial Resistance and Acceptability of Anal Swab Screening: Insights from a Cross-Sectional Study in the General Population and Healthcare Personnel in Argentina

**DOI:** 10.1093/ofid/ofaf695.2250

**Published:** 2026-01-11

**Authors:** Maximiliano Gabriel Castro, María José Sadonio, Melina Tatiana Beloso, Luciano Priotti, Bianca Molaro, Virginia Heinzmann Dotti, Agustín Martínez, Agostina Audicio Schmidt, Ana Paula Amato, Joaquín Portillo, Evangelina Echeverria, Adriel Martínez, Federico Rafael Galluccio, Hector Mario Musacchio

**Affiliations:** CEMIC: Centro de Educacion Medica e Investigaciones Clinicas Norberto Quirno, Ciudad autónoma de Buenos Aires, Ciudad Autonoma de Buenos Aires, Argentina; Hospital Dr. JB Iturraspe, Santa Fe, Santa Fe, Argentina; Hospital Dr. JB Iturraspe, Santa Fe, Santa Fe, Argentina; Hospital Dr. JB Iturraspe, Santa Fe, Santa Fe, Argentina; Hospital Dr. JB Iturraspe, Santa Fe, Santa Fe, Argentina; Hospital Dr. JB Iturraspe, Santa Fe, Santa Fe, Argentina; Hospital Dr. JB Iturraspe, Santa Fe, Santa Fe, Argentina; Hospital Dr. JB Iturraspe, Santa Fe, Santa Fe, Argentina; Hospital Dr. JB Iturraspe, Santa Fe, Santa Fe, Argentina; Hospital Dr. JB Iturraspe, Santa Fe, Santa Fe, Argentina; Faculty of Medical Sciences-National University of the Littoral, Santa Fe, Santa Fe, Argentina; Faculty of Medical Sciences-National University of the Littoral, Santa Fe, Santa Fe, Argentina; Hospital Dr. JB Iturraspe, Santa Fe, Santa Fe, Argentina; Hospital Dr. JB Iturraspe, Santa Fe, Santa Fe, Argentina

## Abstract

**Background:**

Antimicrobial resistance (AMR) is a global health priority. In Argentina, limited data are available regarding public awareness of AMR, as well as the acceptability of specific preventive interventions, such as anal swab (AS) screening. We aimed to assess knowledge and perception of AMR in the general population (GP) and to compare the acceptability of AS between the GP and healthcare workers (HCW) and medical students (MS).

**Figure:**
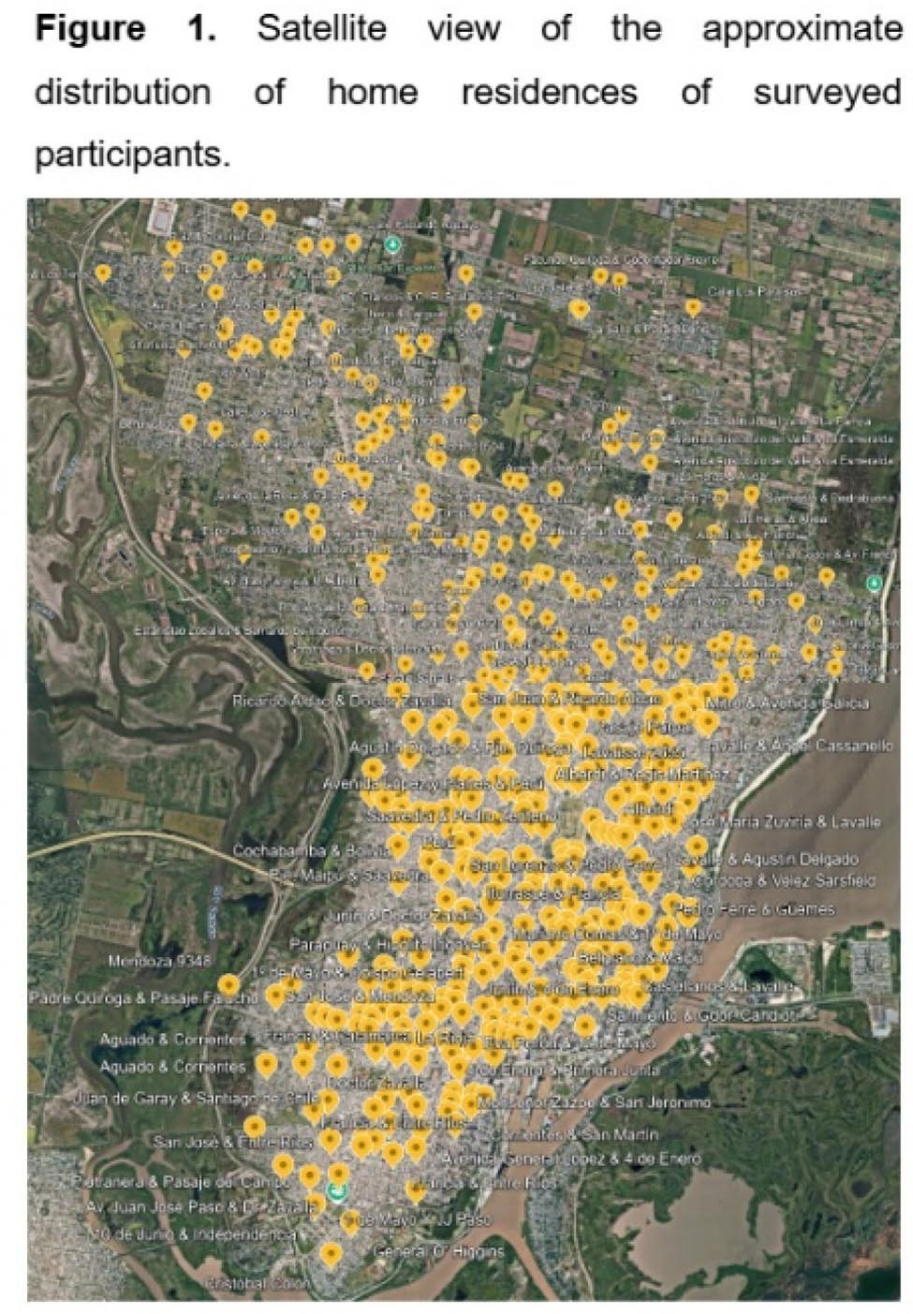

**Figure:**
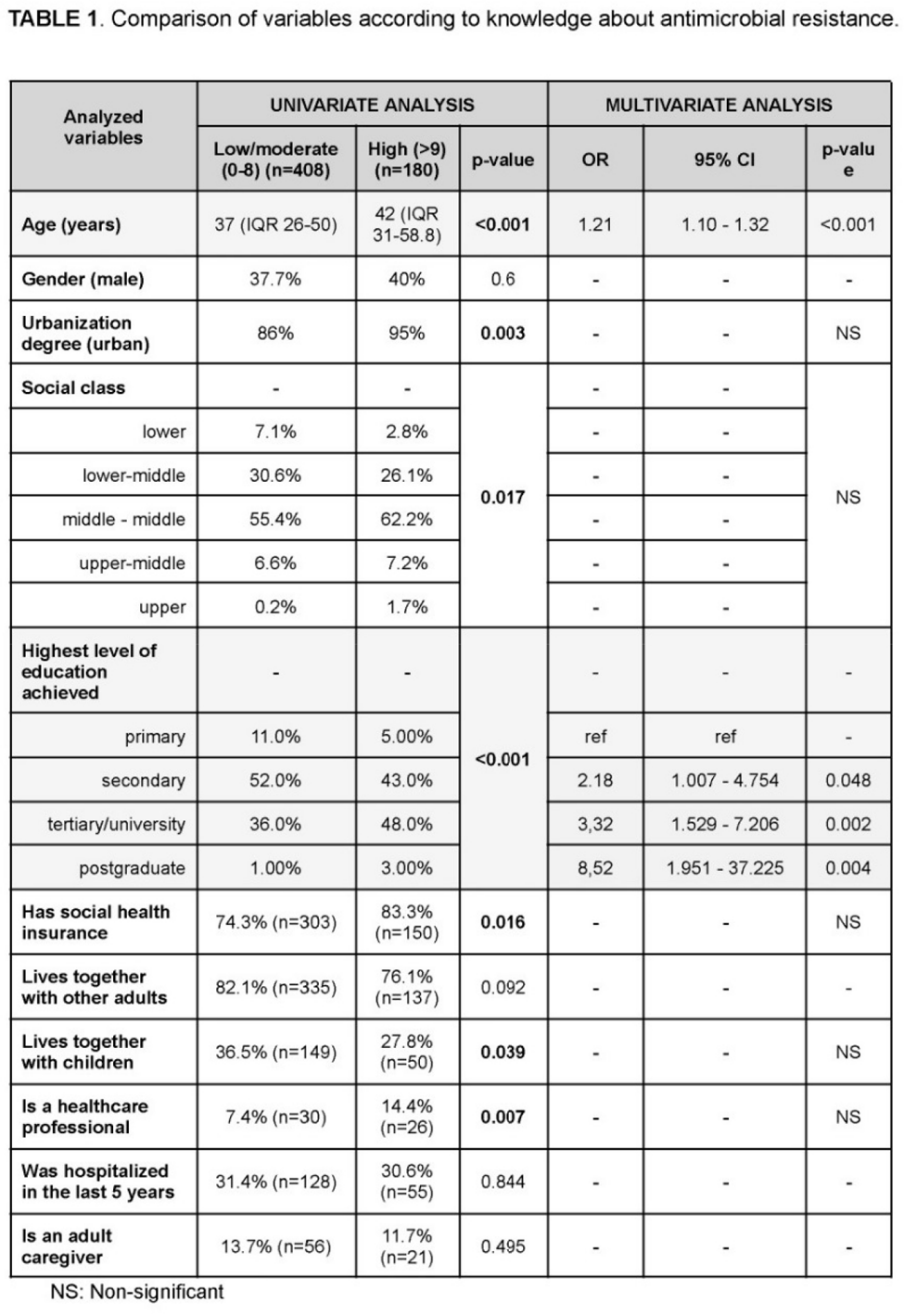

**Methods:**

We conducted a cross-sectional study in the third trimester of 2023 in Santa Fe, Argentina. In Phase 1, an adaptation of the WHO surveys on AMR was administered to the GP during the national presidential elections. In Phase 2, surveys were distributed to MS and HCW in diverse clinical settings. Chi2 and Mann-Whitney’s U test were used for univariate analysis. Variables with a p< 0.05 were included in logistic regression models to predict both AMR knowledge and AS acceptability.

**Figure:**
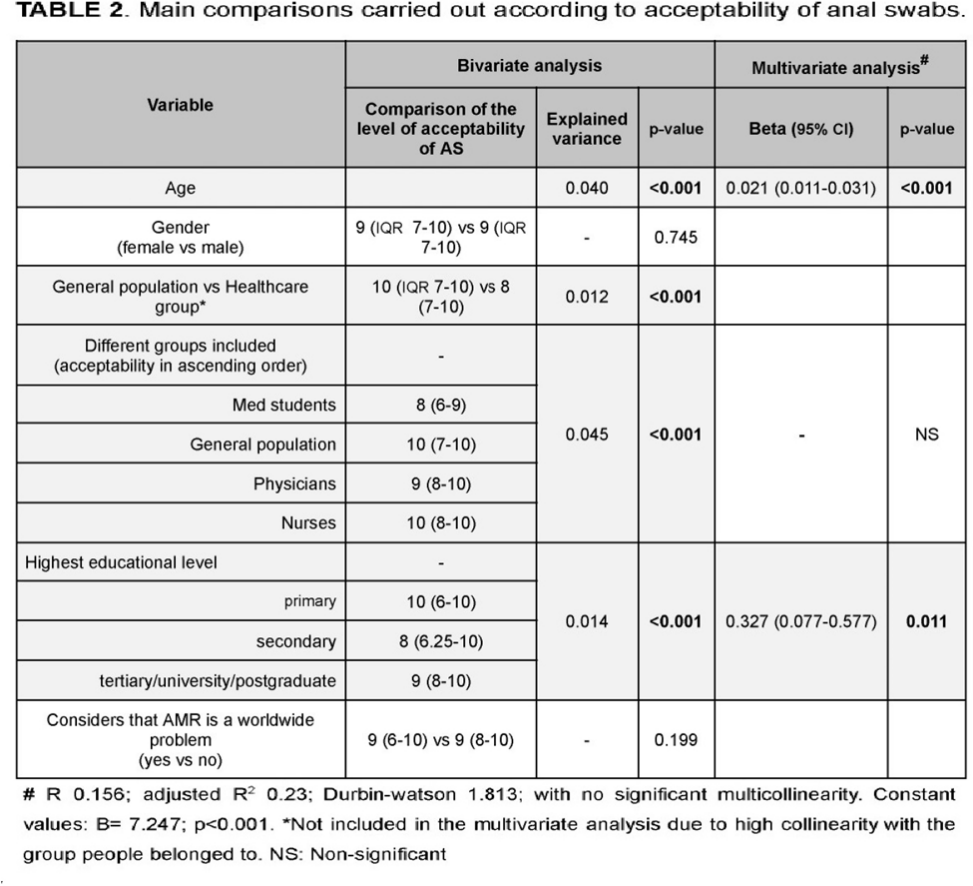

**Results:**

We collected 588 surveys from the GP (Figure 1) and 544 from HCW (28.3% nurses and 23.9% physicians) and MS. In the GP, the median age was 39 years (IQR 27.0–53.8), and 61.1% were female. Most participants (86.8%) identified as middle or lower-middle socioeconomic class. While 46.8% had heard of AMR, only 77.9% of those individuals understood the concept. Knowledge esa high in 30.6%. Factors associated with knowledge in these patients are shown in Table 1. HCW and MS showed lower acceptability of AS compared to the GP, primarily due to lower scores among MS [8 (IQR 6–9) vs. 10 (IQR 7–10); p=0.001]. Factors associated with AS acceptability are shown in Table 2. Among GP, 39.5% thought that AS would be embarrassing for patients, while this was more frequent among physicians (64.6%), nurses (75.3%) and MS (83.9%) (p< 0.001). Self-swabbing was considered acceptable by 35.9% of the GP, compared to 56.2–57.8% of physicians and nurses, and 75.5% of MS (p< 0.001).

**Conclusion:**

Knowledge about AMR in the GP was low to moderate; however, when provided with basic information, the acceptability of AS as a screening measure was high, with a preference for the procedure to be performed by HCW. Among HCW and MS, misconceptions represented barriers to AS implementation. This study contributes to the limited available evidence on the acceptability of infection control measures in both community and clinical settings.

**Disclosures:**

All Authors: No reported disclosures

